# Anatomy of the sternum and humerus in the domestic chicken (*Gallus domesticus*)

**DOI:** 10.1111/vru.13312

**Published:** 2023-11-06

**Authors:** Jeryl C. Jones, Emma E. White, Steven D. Holladay, Jenna L. Foster

**Affiliations:** 1 Department of Animal and Veterinary Sciences, Clemson University, Clemson, South Carolina, USA; 2 South Carolina Translational Research Improving Musculoskeletal Health Center, Clemson University, Clemson, South Carolina, USA; 3 Department of Biomedical Sciences, College of Veterinary Medicine, University of Georgia, Athens, Georgia, USA; 4 Educational Resource Center, College of Veterinary Medicine, University of Georgia, Athens, Georgia, USA

**Keywords:** backyard chickens, egg production, keel bone damage, poultry welfare

## Abstract

Injuries of the sternum and humerus are an important welfare concern in domestic chickens (*Gallus domesticus*), especially laying hens. Published anatomic references using standardized terminology from the *Nomina Anatomica Avium* (*NAA*) are lacking. Objectives of the current retrospective, descriptive study were to provide a user-friendly hierarchical table of *NAA*-compliant anatomic terms and labeled images illustrating anatomic structures for the sternum and humerus of domestic chickens. Three-dimensional model images were downloaded from a publicly accessible platform, labeled in consultation with a veterinary anatomist, and enhanced by a medical illustrator. Findings can serve as a resource for future clinical and research applications.

## 1 | INTRODUCTION

Meat and eggs from domestic chickens (*Gallus domesticus*) are an important source of protein-rich food worldwide.^1,2,3^ Chickens are also increasingly being kept in backyard flocks for egg production or meat animals, or as family pets.^4^ The bones of the sternum and humerus are particularly important because they serve as attachment sites for the major flight muscles in flying species, meat muscles in meat-producing species, and contain extensions of air sac components of the respiratory system.^5^ Injuries in these bones can cause pain, stress, increased susceptibility to disease, reduced activity, reduced productivity, reduced meat quality, and impaired respiratory function.^6,7^ Causes of injury to the sternum in chickens include elevated egg production, poor bone health, and collisions with other hens or solid objects (which may be exacerbated by poor feathering, coarse or wet bedding, or increased time sitting on the sternum).^8^ A user-friendly reference with standardized anatomic terminology for the sternum and humerus will provide a helpful resource for future clinical and research applications in this important animal species.

The current reference standard for avian anatomic terminology worldwide is the *Nomina Anatomica Avium* (*NAA*). However, figures illustrating the anatomy of the sternum and humerus use bones from the domestic pigeon (*Columba livia*) and the European herring gull (*Larus argentatus*), respectively, rather than the chicken.^8^ These species have several noteworthy differences from the chicken, for instance, the presence of a fenestra medialis in the gull sternum as opposed to the incisurae medialis in the chicken sternum. Anatomic terms are also provided only in Latin and described in paragraph format. Getty et al.^5^ provided line drawing figures in their domestic animal anatomy reference book illustrating the anatomy of the chicken sternum and humerus, but often used terminology that was inconsistent with *NAA*. Smallwood published an online avian anatomy book, however, this book did not contain labeled figures of the sternum and humerus as isolated bones.^9^ This text instead featured figures of the complete avian skeleton and most figures were of the domestic turkey (*Meleagris gallopavo domesticus)* rather than the domestic chicken. Koenig et al.^10^ published an avian anatomic atlas that contained color photographs of the sternum and humerus of the domestic chicken. However, some bone structures were not labeled and some were labeled using terms that were inconsistent with *NAA*.

The objectives of the current study were to provide a user-friendly hierarchical table of *NAA*-compliant anatomic terms and labeled images illustrating anatomic structures for the sternum and humerus of domestic chickens.

## 2 | MATERIALS AND METHODS

The study was a retrospective, descriptive design. An undergraduate research student (E.W.) retrieved three-dimensional (3D) model images of the chicken sternum and humerus from a publicly accessible platform for publishing interactive 3D content.^11^ The content creator who scanned and published the bone models provided approval for the use of the images in this study. In consultation with a veterinary anatomist (S.H.) and an ACVR-certified veterinary radiologist (J.J.), the student summarized the *NAA* Latin terminology using a hierarchical table format and inserted Latin and anglicized versions of the *NAA* names for anatomic features into screenshots of the bone models using word document software (Microsoft Word, Microsoft Corp.). A medical illustrator (J.F.) with the Educational Resources department serving the University of Georgia’s College of Veterinary Medicine used these screenshots and labeled references to create more user-friendly images.

Images were imported into image analysis software (Adobe Photoshop 24.5). The images were first retouched using the spot healing brush tool to remove blemishes and a levels adjustment layer was selectively masked in with a feathered brush to soften any hard shadows. The pen tool was then used to create a precise clipping path around the bone’s contour. This path was used to mask the bone and effectively hide any background image data from the screenshot. The bone was then layered above a dark gradient background to provide contrast and clearly define its structure. A hue/saturation adjustment layer was applied to completely desaturate the bone. Next, a solid light-yellow layer was created and applied to the bone layer using the “color” blend mode at 7% opacity to add a natural-looking warm hue back to the bone. A thin white stroke around the bone contour was selectively painted with a feathered brush to mimic a 3D fresnel lighting effect and help clearly define the parts of the bone cast in shadow. A curve adjustment layer was applied to accentuate the bone’s topology.

Once retouched, the bone was rotated per veterinary directional standards. Using the reference provided, labels for the anatomic structures were added in white using the text tool. Leader lines from the descriptors to the data points were also created in white using the pen tool. To improve accessibility, each white leader line was duplicated in place, converted to black, and trimmed with the pen tool to the portion of the line that overlaps the bone so that the leader lines are fully visible on both the dark background and light subject. Additionally, the ellipse tool was used to add a black circle with a thin white stroke to demarcate each specific data point.

An accessible color-coded style system was developed to indicate gross anatomical characteristics of the bones including the body and carina of the sternum, margins, incisures, and anatomical location. The body and carina of the sternum were colorized with a feathered mask overlay. Margins were indicated with a thick dashed line made with the pen tool that was adapted to a line shape with defined stroke properties. A semitransparent outer-glow effect was then applied to the layer. The incisures and anatomical locations were defined by a solid contour stroke and colorized according to the color-coding key. Images were reviewed by all authors, revised as needed, and saved as uncompressed TIF files without layers.

## 3 | RESULTS

**FIGURE 1:**
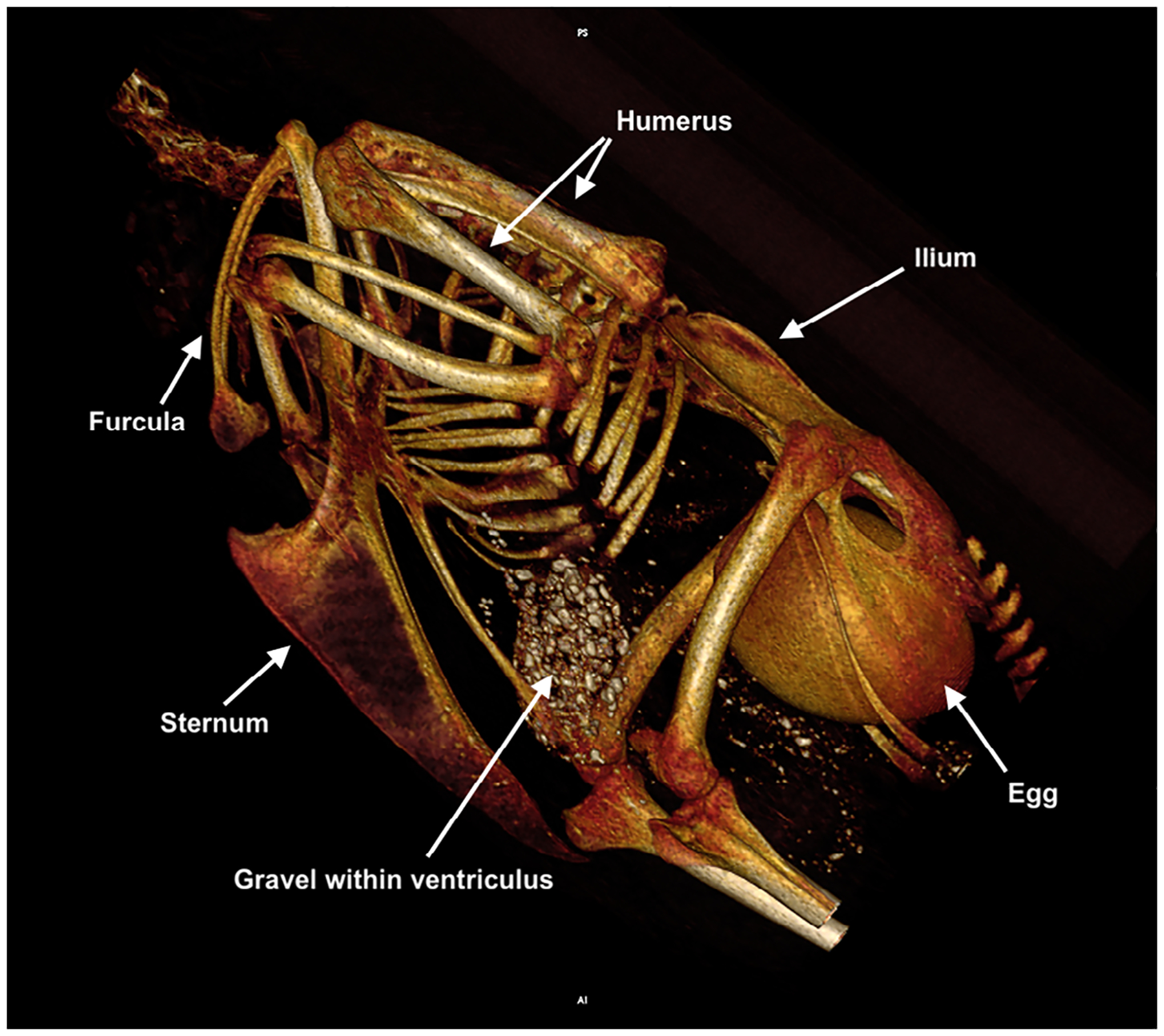
Three-dimensional, volume-rendered, CT image of a mature laying hen illustrating the sternum and humeri in situ, as well as other landmarks (left lateral view). An egg with a well-calcified shell is identified within the pelvic canal.

**FIGURE 2:**
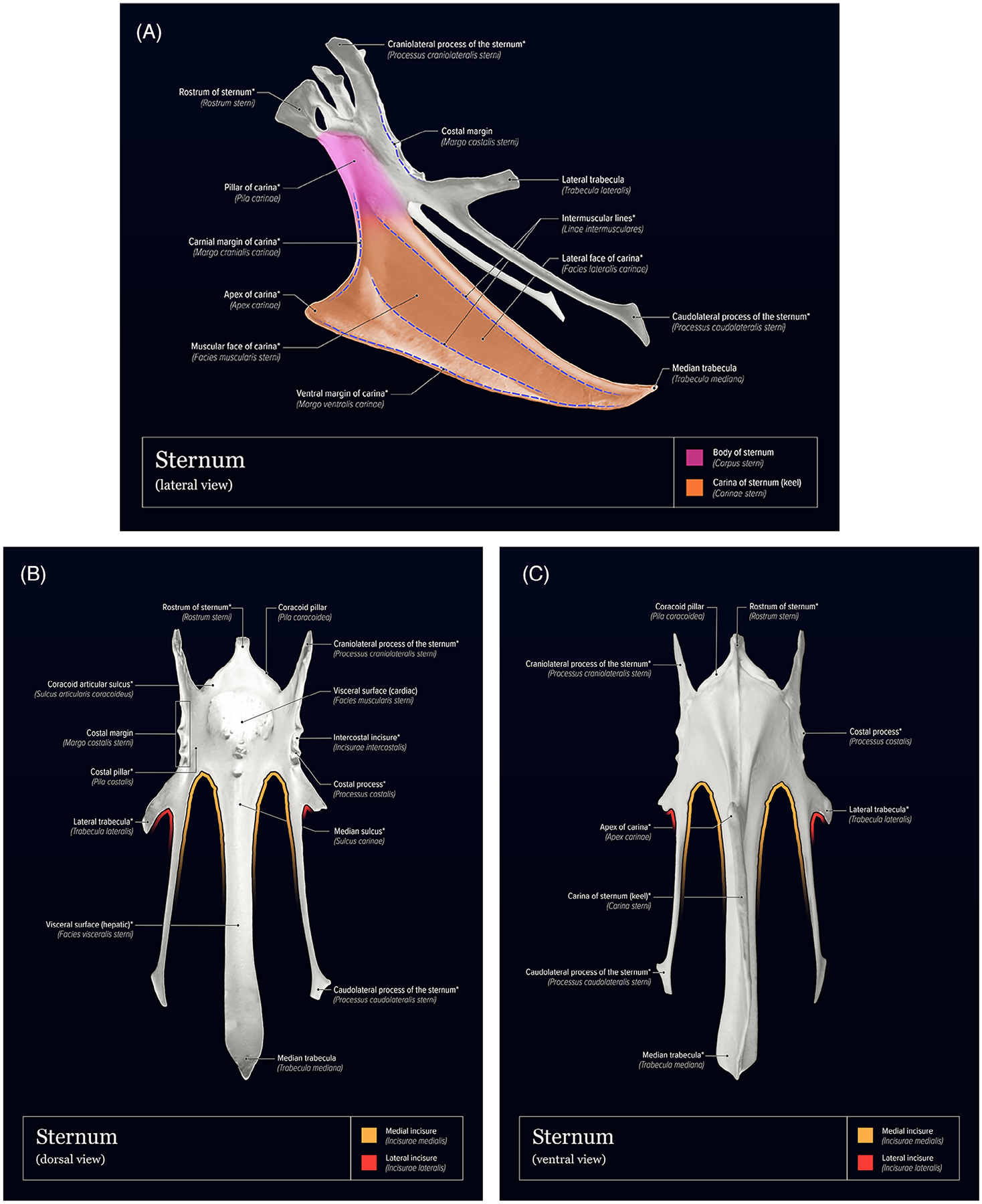
Surface-shaded, labeled images of the domestic chicken sternum. A, Left lateral view; B, dorsal view; C, ventral view. In all images, labels marked with an asterisk (*) represent terms that were not provided or were inconsistent with *Nomina Anatomica Avium* in previously published anatomic reference texts. Images were adapted from 3D bone model images provided by Prof. Sven Reese.^11^

**FIGURE 3:**
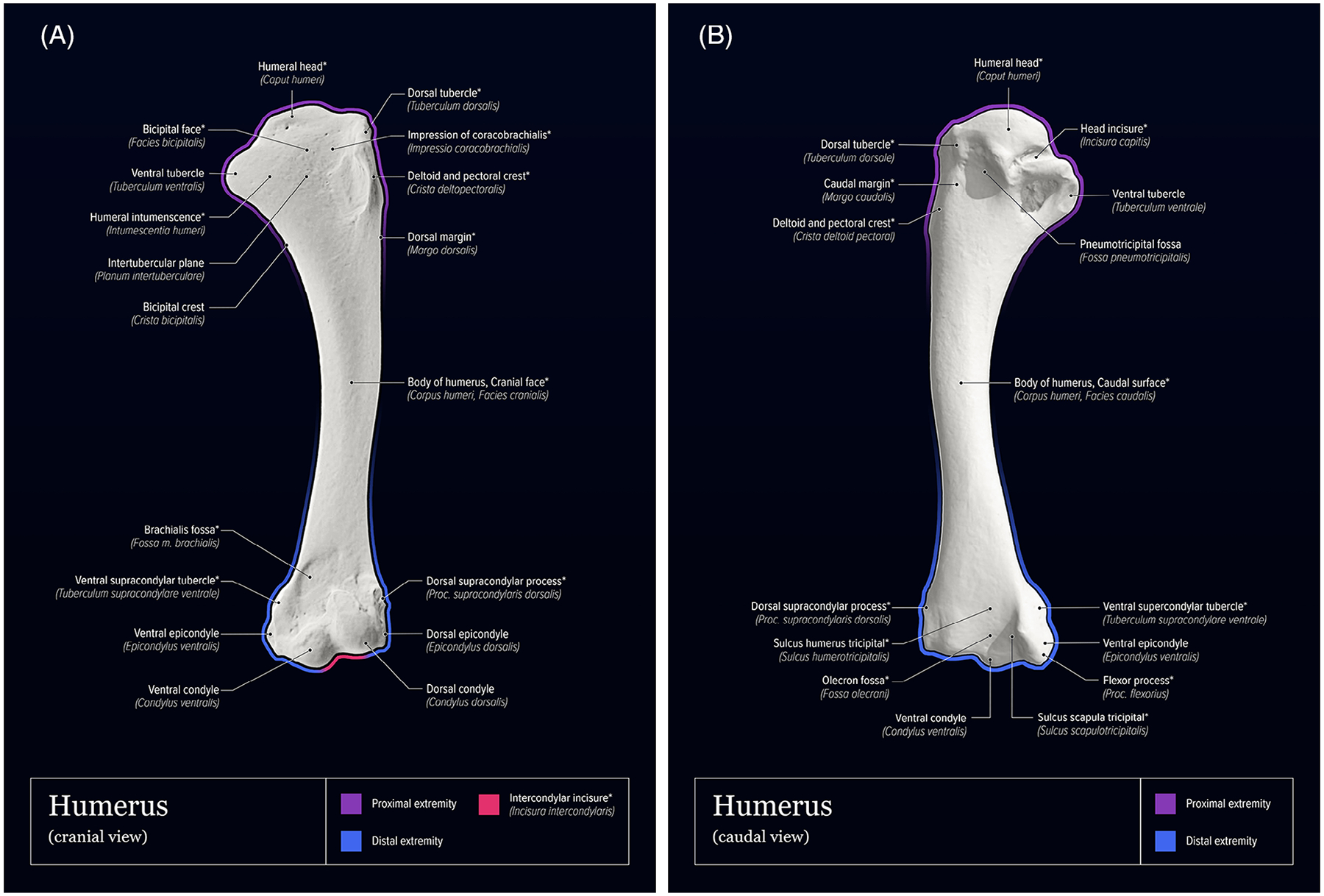
Surface-shaded, labeled images of the domestic chicken humerus. A, Cranial view; B, caudal view. In all images, labels marked with an asterisk (*) represent terms that were not provided or were inconsistent with *Nomina Anatomica Avium* in previously published anatomic reference texts. Images were adapted from 3D bone model images provided by Professor Sven Reese.^11^

Table 1 summarizes Latin anatomic *NAA* terms for components of the avian sternum and humerus, organized hierarchically from whole bone to increasingly finer details. (Table 1). Figure 1 provides a three-dimensional, volume-rendered, CT image example of a mature laying hen illustrating the in-situ relationships of the sternum and humeri. Figures 2–3 provide surface-shaded anatomic images of the individual bones with labels of anatomic features for each bone. Structures that were not named using *NAA* terminology or were not labeled in previously published anatomic reference texts are also identified.

## 4 | DISCUSSION

The current study contributed user-friendly images with detailed, *NAA*-compliant labels of anatomic structures for the chicken humerus and sternum. Images were displayed in a surface-shaded, color-enhanced format. We also organized the *NAA* names for anatomical structures into a table format for quick reference and understanding of how the bones related to one another (general to specific). Findings can be used as a resource for a wide range of users such as veterinary students, primary care veterinarians, veterinary radiologists, veterinary anatomists, animal science students, poultry producers, and poultry researchers.

## Figures and Tables

**TABLE 1:** Latin names from *Nomina Anatomica Avium* for anatomic structures of the domestic chicken sternum and humerus, organized under hierarchical heading categories.

Heading 1	Heading 2	Heading 3	Heading 4	Structure name	Additional structure
Ossa Cinguli Membri Thoracici	Sternum	Corpus sterni	Facies muscularis sterni	Linea intermuscularis	
				Planum postcarinale	
				Pars cadiaca	
				Pars hepatica	
				Foramen pneumaticum	
				Pori pneumatici	
				Sulcus medianus sterni	
			Margo costalis [lateralis] sterni	Incisurae costalis	
				Loculus costalis	
				Pila costalis	
				Proc. Articularis sternocostalis	Facies articularis costalis
			Margo cranialis sterni	Pila coracoidea	
				Sulcus articularis coracoideus	
				Labrum externum	Tuberculum labri externi
				Labrum internum	
				Proc. Craniolateralis sterni	
				Impressio m. sternocoracoidei	
		Rostrum sterni	Foramen rostri		
			Spina externa rostri	Alae spinae sternae	
			Spina interna rostri		
			Spina communis		
			Septum interarticulare		
			Spatium intercoracoidale		
		Margo caudalis sterni	Fenestra lateralis		
			Fenestra medialis		
			Incisura lateralis		
			Trabecula intermedia		
			Trabecula lateralis		
			Trabecula mediana		
			Proc. caudolateralis sterni		
		Carina sterni	Apex carinae		
			Facies articularis furculae		
			Tuberositas lig. sternoclavicularis		
			Facies lateralis carinae	Linea intermuscularis	
			Margo cranialis carinae	Crista lateralis carinae	
				Crista mediana carinae	
				Pila carinae	
				Sulcus carinae	
			Margo ventralis carinae		
Ossa Alae [Membri Thoracici] Skeleton Brachii	Humerus	Extremitas proximalis humeri	Caput humeri	Incisura capitis humeri	
				Crista incisurae capitis	
				Tuberculum dorsale	
			Crista deltopectoralis	Angulus cristae	
				Impressio m. pectoralis	
				Crista m. supracoracoidei	
			Tuberculum ventrale	Crista bicipitalis	
			Fossa pneumotricipitalis [F. tricipitalis]	Foramen pneumaticum	
				Crus dorsale fossae	
				Crus ventral fossae	
				Margo caudalis	
			Planum intertuberculare	Sulcus [Canalis] n. coracobrachialis	
				Intumescentia humeri	
			Sulcus transversus		
			Impressio coracobrachialis		
		Corpus humeri	Facies caudalis	Margo caudalis	
			Facies cranialis	Margo doralis	
					
				Margo ventralis	Linea m. latissimi dorsi
					Sulcus n. radialis
		Extremitas distalis humeri	Condylus dorsalis humeri		
			Condylus ventralis humeri		
			Incisura intercondylaris		
			Fossa m. brachialis		
			Epicondylus dorsalis [Ectepicondylus]		
			Epicondylus ventralis [Ectepicondylus]		
			Proc. flexorius		
			Tuberculum supracondylare dorsale	Proc. supracondylaris dorsalis	
			Tuberculum supracondylare ventrale		
			Fossa olecrani	Sulcus scapulotricipitalis	
				Sulcus humerotricipitalis	
				Os sesamoideum m. scapulotricipitis	

## References

[1] Lesnierowski G, Stangierski J. What’s new in chicken egg research and technology for human health promotion?-A review. Trends Food Sci Technol. 2018;46–51.

[2] Réhault-Godbert S, Guyot N, Nys Y. The golden egg: nutritional value, bioactivities, and emerging benefits for human health. Nutrients. 2019;684. 10.3390/nu11030684 PMC6470839 30909449

[3] Petracci M, Mudalal S, Soglia F, Cavani C, Meat quality in fast-growing broiler chickens. Worlds Poult Sci J. 2015;71(2):363–374.

[4] Elkhoraibi C, Blatchford RA, Pitesky ME, Mench JA. Backyard chickens in the United States: a survey of flock owners. Poult Sci. 2014;2920–2931. 25193256 10.3382/ps.2014-04154

[5] Getty R, Sisson S. 1975; Sisson and Grossman’s the Anatomy of the Domestic Animals. W.B. Saunders.

[6] Toscano MJ, Dunn IC, Christensen J, Petow S, Kittelsen K, Ulrich R. Explanations for keel bone fractures in laying hens: are there explanations in addition to elevated egg production? Poult Sci. 2020;4183–4194. 32867962 10.1016/j.psj.2020.05.035 PMC7597989

[7] Petracci M, Bianchi M, Cavani C. Pre-slaughter handling and slaughtering factors influencing poultry product quality. Worlds Poult Sci J. 2010; 66(1):17–26.

[8] Baumel JJ. Nomina Anatomica Avium: An Annotated Anatomical Dictionary of Birds. Academic Press; 1979;

[9] Smallwood JE. *A* Guided Tou*r of* Birds *and* Their Anato*my*. Guided Tour Books; First ed. 2015;

[10] Koenig HE, Korbel R, Liebich H, Klupiec C. 2016; Avian Anatomy: Textbook and Colour Atlas. 5m Books Ltd.

[11] Reese S vetanatMunich. https://sketchfab.com/vetanatMunich. Updated 2023.

